# Infectious diseases, dividend policy, and independent directors: Evidence from textual analysis

**DOI:** 10.1371/journal.pone.0281109

**Published:** 2023-02-02

**Authors:** Saranyu Ungpakorn, Pattanaporn Chatjuthamard, Pornsit Jiraporn, Piyachart Phiromswad

**Affiliations:** 1 Sasin School of Management, Chulalongkorn University, Bangkok, Thailand; 2 Center of Excellence in Management Research for Corporate Governance & Behavioral Finance, Sasin School of Management, Chulalongkorn University, Bangkok, Thailand; 3 Great Valley School of Graduate Professional Studies, Pennsylvania State University, Malvern, PA, United States of America; 4 Research Unit in Finance and Sustainability in Disruption Era, Sasin School of Management, Chulalongkorn University, Bangkok, Thailand; University of Almeria: Universidad de Almeria, SPAIN

## Abstract

We investigated the effect of uncertainty associated with infectious diseases on corporate dividend policy. We used a unique text-based measure of infectious diseases that includes not only the Covid-19, but also other important diseases, such as SARs, MERs, and Ebola. Based on a sample of 287,151 firm-year observations across four decades (from 1985 to 2021), our results show that a higher level of uncertainty associated with infectious diseases significantly reduce dividends. Interestingly, we also found that having more independent directors on the board mitigates the negative effect of uncertainty associated with infectious diseases on dividends which implies that the reduction in dividends was partly driven by agency conflicts. We performed several robustness checks which confirm that our findings are unlikely to be affected by endogeneity issues.

## I. Introduction

Motivated by the on-going pandemic that has thrown economies and businesses into turmoil, we explored how uncertainty associated with infectious diseases influences corporate dividend policy. While the Covid-19 has obviously dominated the headlines, there have been several other infectious diseases, such as MERS, SARS, and Ebola in the past. Based on a novel text-based measure of uncertainty associated with infectious diseases, we investigated how corporations alter their dividends in response to the emergence of infectious diseases.

In theory, there are two possible hypotheses. First, companies tend to reduce dividends when uncertainty arises. With increased uncertainty, businesses want to reduce dividend payout as they want to conserve financial resources and avoid relying on external financing which could be prohibitively expensive during turbulence times [1–3]. This view suggests a decline in dividends in response to greater uncertainty. By contrast, during uncertain times, agency costs could be more severe [4–8]. Consequently, shareholders demand more dividends with the intent to reduce the free cash flows that otherwise could be exploited by opportunistic managers [8]. This hypothesis predicts that greater uncertainty raises dividends.

Based on a sample of 287,151 firm-year observations that covers more than 30 years of data, our results show that higher uncertainty associated with infectious diseases significantly reduce dividends. Our findings are consistent with the precautionary hypothesis where companies exercise more caution during difficult times and reduce dividends. To demonstrate that our findings are not driven by economic uncertainty in general, we controlled for the degree of economic policy uncertainty (EPU) index proposed by Baker et al. [9]. Our results are qualitatively unaffected by this inclusion which suggests that the effect of uncertainty associated with infectious diseases on dividend policy is above and beyond the effect of economic uncertainty.

Because infectious diseases are evidently beyond any firm’s control, they are likely to be exogenous to firm-specific characteristics. However, uncertainty associated with infectious diseases could be endogenous. Thus, we executed additional robustness checks which include propensity score matching, entropy balancing, dynamic panel data analysis with over-identifying restriction tests, and Oster’s [10] approach for testing coefficient stability. All these robustness checks validate our results.

Furthermore, we examined how corporate governance influences the effect of uncertainty associated with infectious diseases on corporate dividends. We concentrated on board independence since it is one of the most important mechanism for corporate governance. Our results show that having more independent directors reduces the effect of uncertainty associated with infectious diseases on corporate dividends. This implies that the effect of uncertainty associated with infectious diseases on corporate dividends is partly driven by agency problems which can be mitigated by more effective governance.

The results of our study provide several contributions to the literature. First, we contribute to the literature on corporate dividend policy [11–17]. We loosen the assumption of perfect capital markets proposed by Modigliani and Miller [18] by incorporating a market imperfection in the form of uncertainty associated with infectious diseases that has not been examined in the literature. Second, our study contributes to a timely and important area of the literature that focuses on the effects of infectious diseases [19–21]. Although previous studies concentrate on the effects of infectious diseases on macroeconomic outcomes, studies on the effects of infectious diseases on specific corporate policies and outcomes are sparse. Our study is the first to demonstrate that uncertainty associated with infectious diseases has a significant effect on corporate dividend policy. Moreover, our findings contribute to a large body of knowledge that investigates the effect of corporate governance on dividend policy [4,22–27]. We found that having more independent directors reduces the effect of uncertainty associated with infectious diseases on corporate dividends. As far as we are aware, our study is the first to link board governance to dividend policy during uncertain times. Finally, our research adds to a growing body of work that employs textual analysis and machine learning [28–36]. Loughran and McDonald [37] go over the applications of textual analysis in accounting and finance in detail. We expect this novel measure to be more frequently used in the future.

## II. Literature review and development of hypotheses

### a. Dividend policy and uncertainty

Uncertainty can have an impact on business choices because higher uncertainty reduces managers’ ability to foresee firm-specific financial indicators such as predicted future cash flows [3,38]. Prior research has documented the effects of economic policy uncertainty on various aspects of corporate outcomes and managerial decisions such as R&D expenditures [39,40], merger and acquisition [41,42], executive risk-taking incentives [43], innovation [44], equity prices and risk premiums [45], and financial reporting quality [3].

In recent years, there has been a surge in research on the role of uncertainty on dividend policy motivated by the financial crisis of 2008 and the worldwide economic slump [8]. For instance, Abreu and Gulamhussen [46], Bliss et al. [47], and Attig et al. [48] demonstrated that corporates modify their dividend policy in reaction to crises. Likewise, Farooq and Ahmed [49] recently documented that companies pay more dividends during presidential election years compared to non-election years as elections are associated with more uncertainty [8]. Finally, it has been reported that a higher level of economic policy uncertainty is associated with higher dividend payouts which is intended to mitigate agency problems when firms experience economic policy uncertainty [8].

### b. The role of infectious disease on corporate decisions

Several studies found that the perception of managers regarding the uncertainty of future earnings can affect corporate dividend policy [1,50,51]. Other studies found that firms have to adjust their corporate’s policy such as corporate investment [52–54] and corporate dividend payout policy [55] in order to survive in the Covid-19 pandemic period. Suleman and Yaghoubi [56] studied the effect of pandemics through infectious disease equity market volatility index on corporation actions. They showed that firms decrease their corporate investment, debt maturity, debt levels, and dividend payment during pandemics. On the other hand, they found that firms increases their of cash holding and corporate investment in research and development (R&D) during pandemics.

### c. Hypothesis development

It is undeniable that the latest pandemic has affected economies worldwide. While the Covid-19 pandemic has received the most attention within the research community, there have been several other notable infectious diseases in the past few decades, such as MERS, SARS, and Ebola. Furthermore, while there are prior research studies that investigate the effects of economic uncertainty on corporate policies in general, there has been limited research done on the effect of uncertainty associated with infectious diseases. To fill these literature gaps, we examined the effect of uncertainty associated with all types of infectious diseases on corporate dividend policy.

According to the literature, the uncertainty associated with infectious diseases is different from other types of economic uncertainty. First, unlike the uncertainty associated with economic and political factors (such as the global financial crisis of 2008 as well as the presidential or gubernatorial elections) which tend to affect only the demand side of the economy, uncertainty associated with infectious diseases such as the Covid-19 pandemic can affect both the demand as well as the supply side of the economy [57–59]. For example, a pandemic can affect the production process (as several manufacturing plants are shutdown due to infection of their workers) and disrupt supply chain process (as several borders are close or become more restricted) globally. Second, pandemics are more difficult to resolve from the policy makers’ perspective. For financial or economic crises, policy makers would normally use fiscal and monetary policies to resolve problems stemmed from the demand side of the economy. However, coping with a pandemic-related crisis is different and much more challenging as policy makers would have to coordinate many policies from vastly distinct government entities such lockdowns, quarantines, and social distancing. These are beyond the typical financial and economic policies.

Based on the literature, we proposed two hypotheses regarding the effect of uncertainty associated with infectious diseases on dividend policy. First, firms reduce dividend payouts when uncertainty associated with infectious diseases increases. When uncertainty increases, this could affect investors’ risk perception [3,45] which can increase the cost of capital and reducing firms’ ability to payout dividends. Furthermore, firms may want to be more cautious and conserve financial resources by reducing dividend payout. This will allow firms to have enough financial resources during turbulence times and do not need to rely on external financing which can be prohibitively expensive [1–3]. We refer to this view as the precautionary hypothesis [3].

Second, firms increase dividend payouts when uncertainty associated with infectious diseases increases. As a way of dealing with the free cash flow problem, shareholders demand more dividend payouts during uncertain times because agency conflicts are worsened [4–8]. Higher uncertainty tend to induce firms to increase monetary reserves which can be easily converted into private benefits [8,60]. Furthermore, higher uncertainty means that it is more difficult to predict future returns. Thus, shareholders may prefer to receive more dividend payouts now rather than having firms making investments on their behalf for future returns. We refer to this view as the agency cost hypothesis [3].

### d. The role of independent directors

In addition, we explored the role of corporate governance on the effect of uncertainty associated with infectious diseases on dividend policy. Several studies examine the effect of corporate governance on corporate dividend policy [4,22–26]. As the board of directors is often considered as a key governance mechanism, we chose to examine the role of board independence in this study. We focused on the percentage of independent directors on the board since they are less dependence on the firm which should them be less biased. Thus, this will lead to more effective corporate governance. Several studies demonstrates the role of independent directors in corporate governance. Rosenstein and Wyatt [61] documented that when independent directors are appointed, the stock market responds favorably. Cotter, Shivdasani, and Zenner [62] studied the role of independent directors in mergers and acquisitions. They discovered that independent directors increase target shareholder returns from tender offers. Core, Holthausen, and Larcker [63] found a positive relationship between the percentage of independent directors and the market-to-book ratio. Nguyen and Nielsen [64] demonstrated a significant decline in the stock price when independent directors die. Jenwittayaroje and Jiraporn [65] examined the impact of independent directors on corporate performance during the 2008 financial crisis. They found that independence directors had positive effect on firm performance during the crisis period.

In our context and based on agency theory, self-interested managers are less motivated to pay dividend since this will reduce the free cash flow that they could otherwise use for their own benefits [27]. This possibility is even more likely in the periods of pandemic since managers could use the presence of pandemic as an excuse to increase free cash flow by reducing dividends for their own benefits. Thus, a more effective governance mechanism in the form of stronger board independence should mitigate these agency problems.

## III. Sample selection and data description

### a. Sample selection

We focused on firms in the United States. We obtained dividends data and firm characteristics which are used as controlled variables from COMPUSTAT. The data on the infectious disease equity market volatility index are from Baker et al. [9]. We obtained the data on board characteristics (only available from 1996 to 2019) from The Institutional Shareholder Services (ISS). Outliers are excluded at the 1% and 99% levels. In total, we have 287,151 firm-year observations across four decades (from 1985 to 2021).

### b. Uncertainty induced by infectious diseases

Our measure of disease-related uncertainty is the infectious disease equity market volatility index developed by Baker et al. [9]. Using textual analysis, Baker et al. [9] searched for news articles related to infectious diseases and equity market volatility. A higher fraction of these news articles to all articles in each period signifies a higher level of market uncertainty that can be attributed to infectious diseases. For infectious diseases, they identified news articles that use the phrases “epidemic”, “pandemic”, “virus”, “flu”, “illness”, “corona virus”, “MERs”, “SARs”, “Ebola”, “H5N1”, or “H1N1”. See Baker et al. [9] for more detail about the construction of the index. Not surprisingly, the Covid-19 pandemic has made this index gain more attention in the literature and has been utilized extensively in several recent studies [66–68].

### c. Dividend payouts and other variables

As primary measure of our dividend payouts, we used dividends to total assets. As robustness check, we also adopted two alternative measures which are dividends to sales and dividends to net income. These are common measures used in the literature to capture corporate dividend policy. We also incorporated a number of control variables that may affect dividends which are presented in Appendix A1 in S1 Appendix. These include firm size, profitability (measured as by the ratio of EBIT to total assets), leverage ratio, capital expenditures to total assets, the ratio of research and development (R&D) to total assets, the ratio of advertising expenditure to total assets, the ratio of cash holdings to total assets, the ratio of stock repurchases to total assets and discretionary spending. To ensure that our results are not driven by the level of economic uncertainty, we included the Economic Policy Uncertainty Index (EPU) developed by Baker, Bloom, and Davis [36] as another control variable. To capture unobserved variation across industries, we included industry fixed effects based on the first two digits of the SIC codes. Table 1 presents the descriptive statistics for all variables in this study.

**Table 1:** Descriptive statistics. Our measure of disease-related uncertainty is the infectious disease equity market volatility developed by Baker et al. (2020) [9]. Using sophisticated textual analysis, Baker et al. (2020) [9] search for news articles related to infectious diseases and equity market volatility. A higher fraction of these news articles to all articles in each time period signifies a higher level of market uncertainty that can be ascribed to infectious diseases. For ease of interpretation, we have the infectious disease equity market volatility index divided by 100.

	Mean	S.D.	25th	Median	75th
Dividends					
Dividends/Total Assets	0.018	0.052	0.000	0.000	0.012
Dividends/Sales	0.692	82.285	0.000	0.000	0.015
Dividends/Net Income	0.209	11.529	0.000	0.000	0.106
Infectious Disease Index					
Infectious Disease Index	0.010	0.033	0.003	0.005	0.006
Firm-specific Characteristics					
Total Assets	3433.303	28000.000	17.314	110.985	772.272
Total Leverage/Total Assets	0.351	0.667	0.026	0.217	0.417
EBIT/Total Assets	-0.237	1.154	-0.102	0.040	0.099
Capital Expenditures/Total Assets	0.071	0.833	0.012	0.035	0.077
Advertising Expense/Total Assets	0.010	0.032	0.000	0.000	0.002
R&D Expense/Total Assets	0.063	0.170	0.000	0.000	0.038
Cash Holdings/Total Assets	0.197	0.247	0.022	0.087	0.276
SG&A Expense/Total Assets	0.413	0.901	0.034	0.181	0.424
Repurchase/Total Assets	0.009	0.030	0.000	0.000	0.001
Economic Policy Uncertainty Index	108.405	32.379	82.054	107.766	117.582
Board Attributes					
% Independent Directors	71.970	17.037	62.500	75.000	85.714
Board Size	9.147	2.320	7.000	9.000	11.000

## IV. Results

### Baseline regression analysis

The results of the baseline regression analysis are shown in Table 2 Panel A where the dependent variable is the ratio of dividends to total assets. Model 1 and Model 2 are an OLS and a Tobit regression respectively. Because dividends cannot be below zero, they can be viewed as a censored variable and a Tobit regression would be an interesting analysis. The coefficients of the infectious disease index are both negative and significant in Model 1 and Model 2. In Model 3 and Model 4, we added all the control variables including the level of economic uncertainty. Again, the coefficients of the infectious disease index remain significantly negative. Thus, the results of the aselyne analysis are consistent with the precautionary hypothesis even after controlling for the level of economic uncertainty.

**Table 2:** The effect of infectious diseases on corporate dividend policy. Our measure of disease-related uncertainty is the infectious disease equity market volatility developed by Baker et al. (2020) [9]. Using sophisticated textual analysis, Baker et al. (2020) [9] search for news articles related to infectious diseases and equity market volatility. A higher fraction of these news articles to all articles in each time period signifies a higher level of market uncertainty that can be ascribed to infectious diseases. For ease of interpretation, we have the infectious disease equity market volatility index divided by 100.

**Panel A: Baseline Regression analysis**				
	(1)	(2)	(3)	(4)
	OLS	Tobit	OLS	Tobit
	Dividend/Total Assets	Dividend/Total Assets	Dividend/Total Assets	Dividend/Total Assets
				
**Infectious Disease Index**	-0.010***	-0.041***	-0.015***	-0.067***
	**(-3.978)**	**(-6.015)**	**(-3.808)**	**(-7.483)**
Ln (Total Assets)			0.001***	0.011***
			(9.802)	(111.963)
Leverage			0.002***	0.008***
			(3.030)	(16.841)
Profitability			0.001***	0.003***
			(3.377)	(6.045)
Capital Investments			-0.000	-0.007***
			(-0.752)	(-6.206)
Advertising Intensity			-0.006	-0.031***
			(-1.002)	(-4.100)
R&D Intensity			0.016***	0.014***
			(8.763)	(7.885)
Cash Holdings			0.002**	-0.037***
			(2.039)	(-31.439)
Discretionary Spending			0.003***	0.004***
			(5.347)	(7.992)
Share Repurchases			0.063***	0.190***
			(11.739)	(27.048)
EPU Index			-0.000	-0.000***
			(-0.235)	(-5.424)
Constant	0.018***	-0.042***	0.010***	-0.096***
	(80.945)	(-151.028)	(13.528)	(-81.647)
Industry Fixed Effects	Yes	Yes	Yes	Yes
Observations	287,150	287,151	287,150	287,151
Adjusted R-squared	0.026		0.032	
**Panel B: Baseline Regression analysis with the alternative measures of dividends**				
			(1)	(2)
			Dividends/Sales	Dividends/Net Income
		
**Infectious Disease Index**			-87.769***	-12.685***
			**(-6.843)**	**(-6.326)**
Ln (Total Assets)			13.467***	1.500***
			(87.808)	(61.430)
Leverage			-7.939***	-1.018***
			(-8.987)	(-4.867)
Profitability			-3.120***	1.711***
			(-3.863)	(5.207)
Capital Investments			-1.292	-6.609***
			(-0.874)	(-8.858)
Advertising Intensity			-0.065	-5.535***
			(-0.006)	(-2.933)
R&D Intensity			-25.288***	-23.199***
			(-7.373)	(-17.570)
Cash Holdings			-55.841***	-5.617***
			(-28.836)	(-16.951)
Discretionary Spending			2.057**	0.635**
			(2.214)	(2.561)
Share Repurchases/Sales			134.897***	
			(23.331)	
EPU Index			0.001	0.012***
			(0.103)	(5.980)
Share Repurchases/Net Income				1.411***
				(16.575)
Constant			-129.947***	-13.242***
			(-20.265)	(-13.613)
Industry Fixed Effects			Yes	Yes
Observations			261,777	158,244
**Panel C: Baseline Regression analysis with additional control variables**				
			(1)	(2)
			OLS	Tobit
			Dividend/Total Assets	Dividend/Total Assets
		
**Infectious Disease Index**			-0.0128***	-0.0542***
			**(-3.2542)**	**(-6.5884)**
Ln (Total Assets)			0.0009***	0.0095***
			(9.4682)	(91.6876)
Leverage			0.0016**	0.0025***
			(2.4418)	(5.2801)
Profitability			0.0007	0.0020***
			(1.5266)	(4.0489)
Capital Investments			-0.0001	-0.0012
			(-0.8277)	(-1.4906)
Advertising Intensity			0.0028	-0.0120
			(0.4034)	(-1.5598)
R&D Intensity			0.0135***	0.0183***
			(6.5218)	(9.5957)
Cash Holdings			0.0034***	-0.0224***
			(2.5823)	(-18.8514)
Discretionary Spending			0.0021***	0.0075***
			(3.9590)	(13.4019)
Share Repurchases/Total Assets			0.0660***	0.1894***
			(12.2344)	(28.3126)
GDP Growth Rate			0.0001**	0.0001
			(2.5345)	(1.0322)
Change in the Unemployment Rate			0.0002	-0.0007**
			(1.0850)	(-2.4823)
Change in the Consumer Price Index			0.0002**	-0.0000
			(2.2365)	(-0.2136)
Constant			0.0091***	-0.0762***
			(15.9921)	(-17.2273)
Industry Fixed Effects			Yes	Yes
Observations			256,736	256,736
Adjusted/Pseudo R-squared			0.036	-1.436

Robust t-statistics in parentheses.

t-statistics in parentheses.

*** p<0.01

** p<0.05

* p<0.1.

We estimated the economic significance of the uncertainty associated with infectious diseases on dividends as follows. The coefficient of the infectious disease index in shown in Model 4 is -0.067. A one standard deviation of the infectious disease index is 0.033. So, a rise in the infectious disease index by one standard deviation reduces dividends by 0.033 times 0.067, which is 0.001. In our sample, the dividends to total assets for average firms is 0.018. A drop of dividends by 0.001 is equivalent to a 5.55% decline. Therefore, the effect documented here is not only statistically significant, but it is also economically important as well. We also confirmed the results using alternative measures of dividends in Table 2 Panel B. Specifically, we use dividends to sales and dividends to net income. The results are qualitatively unaffected and reinforce the precautionary hypothesis.

Next, we controlled for macroeconomic conditions. In particular, we included a number of macroeconomic variables, namely the GDP growth rate, change in the unemployment rate, and change in the consumer price index (CPI). These variables capture macroeconomic conditions which could affect corporate dividend policy. The regression results are shown in Table 2 Panel C. The coefficients of the infectious disease index are negative and significant in both regressions. Thus, even after we have accounted for macroeconomic conditions, our main results remain robust.

Furthermore, we included VIX or the CBOE volatility index to control for managers’ expectation of volatility. A major advantage of this index is that it is a market-based forward looking index, i.e., it is based on the implied volatility of options traded. Hence, it is different from the other control variables we have used so far, which are either contemporaneous or backward-looking. The regression result is displayed in Table 3. The coefficient of the infectious disease index remains negative and significant.

**Table 3:** Regression analysis controlling for VIX (CBOE volatility index). Our measure of disease-related uncertainty is the infectious disease equity market volatility developed by Baker et al. (2020) [9]. Using sophisticated textual analysis, Baker et al. (2020) [9] search for news articles related to infectious diseases and equity market volatility. A higher fraction of these news articles to all articles in each time period signifies a higher level of market uncertainty that can be ascribed to infectious diseases. For ease of interpretation, we have the infectious disease equity market volatility index divided by 100.

	(1)	(2)
	Dividends/Total Assets	Dividends/Total Assets
		
**Infectious Disease Index**	-0.018***	-0.069***
	**(-5.853)**	**(-8.881)**
Ln (Total Assets)	0.001***	0.010***
	(5.780)	(82.370)
Leverage	0.002***	0.004***
	(3.207)	(7.796)
Profitability	0.002***	0.005***
	(3.617)	(9.141)
Capital Investments	-0.000	-0.001
	(-0.499)	(-1.196)
Advertising Intensity	-0.004	-0.030***
	(-0.618)	(-3.363)
R&D Intensity	0.017***	0.032***
	(8.916)	(16.048)
Cash Holdings	0.001	-0.029***
	(1.117)	(-21.818)
Discretionary Spending	0.002***	0.009***
	(4.614)	(14.829)
Share Repurchases/Total Assets	0.066***	0.207***
	(11.380)	(26.740)
VIX Index	0.000*	-0.000**
	(1.898)	(-2.166)
Constant	0.011***	-0.083***
	(14.447)	(-15.811)
Industry Fixed Effects	Yes	Yes
Observations	251,208	251,208
Adjusted/Pseudo R-squared	0.030	3.518

Robust t-statistics in parentheses.

*** p<0.01

** p<0.05

* p<0.1.

Finally, we considered a firm fixed effect analysis since it should help accounting for unobservable variables that stay constant through time. The regression result is displayed in Table 4. We found that the coefficient of the infectious disease index is not statistically significant. One plausible explanation could be that the fixed effect analysis might not be appropriated in our context because there is little variation of dividend policy over time. This implies that dividend policy tends to be sticky in the short run. To examine this, we computed the standard deviations of the dividends to total assets ratio both across firms and across time and find that the variance between firms is around 67 percent larger than the variation within firms which supports our assumption.

**Table 4:** Regression analysis on fixed effect model. Our measure of disease-related uncertainty is the infectious disease equity market volatility developed by Baker et al. (2020) [9]. Using sophisticated textual analysis, Baker et al. (2020) [9] search for news articles related to infectious diseases and equity market volatility. A higher fraction of these news articles to all articles in each time period signifies a higher level of market uncertainty that can be ascribed to infectious diseases. For ease of interpretation, we have the infectious disease equity market volatility index divided by 100.

	Fixed Effect Model	
	Dividends/Total Assets	Dividends/Total Assets
		
**Infectious Disease Index**	**0.004**	**0.334**
** **	**(1.008)**	**(0.418)**
Ln (Total Assets)	-0.002***	-0.027
	(-13.574)	(-0.765)
Leverage	0.001*	0.502***
	(1.699)	(3.500)
Profitability	0.001**	-0.558***
	(1.988)	(-4.990)
Capital Investments	-0.000	-0.422*
	(-0.224)	(-1.873)
Advertising Intensity	0.003	-1.201
	(0.336)	(-0.655)
R&D Intensity	0.010***	-0.162
	(5.814)	(-0.186)
Cash Holdings	0.003***	-0.314
	(2.983)	(-0.724)
Discretionary Spending	0.001***	-0.306***
	(2.988)	(-2.646)
Share Repurchases	0.048***	-1.251***
	(10.985)	(-3.202)
EPU Index	-0.000	0.000
	(-0.137)	(0.215)
Constant	0.026***	0.918***
	(24.959)	(3.898)
Firm Fixed Effects	Yes	Yes
Observations	285,728	154,990
Adjusted R-squared	0.455	0.044

Robust t-statistics in parentheses.

*** p<0.01

** p<0.05

* p<0.1.

### b. Propensity score matching (PSM) and entropy balancing

In this section, we used the propensity score matching method to strengthen our analysis. Several studies [69–74] adopted this method as it could be used to alleviate omitted variables bias. We divided the samples into quartiles based on the infectious disease index. One group of observations is denoted as the “treatment” group and consists of observations from the upper quartile. Then, using nine firm characteristics, we chose the most comparable observations from the remaining samples for each observation in the treatment group. As a result, our treatment and control firms should be nearly identical in observable firm characteristics except their score in the infectious disease index.

We ran diagnostic tests to check the validity of our matching. Table 5 Panel A summarizes the findings. Model 1 is a logistic regression with a binary dependent variable equal to one if the firm is in the treatment group and zero otherwise. Model 1 includes all observations. The result shows that the treatment firms differ substantially from other non-treatment firms. In particular, the treatment firms are larger in size, more leveraged, less profitable, invest less in advertising, invest more in R&D, and hold more cash.

**Table 5:** Propensity score matching (PSM). Our measure of disease-related uncertainty is the infectious disease equity market volatility developed by Baker et al. (2020) [9]. Using sophisticated textual analysis, Baker et al. (2020) [9] search for news articles related to infectious diseases and equity market volatility. A higher fraction of these news articles to all articles in each time period signifies a higher level of market uncertainty that can be ascribed to infectious diseases. For ease of interpretation, we have the infectious disease equity market volatility index divided by 100.

Panel A: Diagnostic testing		
	(1)	(2)
	Pre-Match	Post-Match
	Treatment (High Infectious Disease Index)	Treatment (High Infectious Disease Index)
		
Ln (Total Assets)	0.074***	-0.003
	(42.878)	(-1.322)
Leverage	0.013*	0.003
	(1.731)	(0.301)
Profitability	-0.087***	-0.016
	(-13.038)	(-1.634)
Capital Investments	-0.029	-0.013
	(-1.073)	(-0.723)
Advertising Intensity	-1.473***	0.006
	(-9.289)	(0.028)
R&D Intensity	0.162***	0.026
	(5.917)	(0.646)
Cash Holdings	0.329***	-0.036
	(17.877)	(-1.292)
Discretionary Spending	0.007	-0.002
	(0.885)	(-0.148)
Share Repurchases	0.048	0.150
	(0.355)	(0.788)
Constant	-1.659***	-0.111
	(-20.437)	(-0.884)
Industry Fixed Effects	Yes	Yes
Pseudo R-squared	0.007	0.000
Observations	287,150	133,358
**Panel B: The effect of infectious diseases on corporate dividend policy**		
	(1)	(2)
	OLS	Tobit
	Dividends/Total Assets	Dividends/Total Assets
		
**Infectious Disease Index**	-0.019***	-0.050***
	**(-3.398)**	**(-4.276)**
Ln (Total Assets)	0.001***	0.010***
	(5.171)	(59.535)
Leverage	0.002***	0.004***
	(2.947)	(6.210)
Profitability	0.001	0.002**
	(1.128)	(2.515)
Capital Investments	0.001	0.000
	(0.540)	(0.346)
Advertising Intensity	0.000	-0.019
	(0.007)	(-1.559)
R&D Intensity	0.019***	0.035***
	(8.162)	(13.470)
Cash Holdings	-0.000	-0.032***
	(-0.144)	(-18.157)
Discretionary Spending	0.001**	0.006***
	(2.119)	(7.707)
Share Repurchases	0.065***	0.202***
	(8.661)	(19.472)
EPU Index	0.000	-0.000***
	(0.724)	(-2.964)
Constant	0.012***	-0.077***
	(11.429)	(-11.175)
Industry Fixed Effects	Yes	Yes
Observations	133,358	133,358
Adjusted/Pseudo R-squared	0.030	0.375

Robust z-statistics in parentheses.

*** p<0.01

** p<0.05

* p<0.1.

Robust t-statistics in parentheses.

Model 2 is a logistic regression that includes only the matched observations. The coefficients in Model 2 are statistically insignificant which implies that the treatment and the control are statistically identical for observable characteristics. The regression results for the propensity score matched sample are shown in Table 5 Panel B. The coefficient of the infectious disease index is negative and significant implying that uncertainty associated with infectious diseases reduces dividend payouts and supporting the precautionary hypothesis once more.

However, propensity score matching might not be able to ensure that all of the moments (e.g., mean) of the treatment and non-treatment groups are exactly identical. An alternative and more modern method called entropy balancing has been proposed and gaining popularity in the literature [71,75–88]. The key idea of the entropy balancing is to incorporate a set of chosen moments into the optimization process in determining the optimal weights for choosing the treatment and non-treatment observations. This approach will make the treatment and non-treatment observations more well-balanced [77] and improve power of testing statistics [78]. Table 6 displays the regression results based on the entropy balancing method. The coefficient of the infectious disease index remains negative and significant.

**Table 6:** Entropy balancing. Our measure of disease-related uncertainty is the infectious disease equity market volatility developed by Baker et al. (2020) [9]. Using sophisticated textual analysis, Baker et al. (2020) [9] search for news articles related to infectious diseases and equity market volatility. A higher fraction of these news articles to all articles in each time period signifies a higher level of market uncertainty that can be ascribed to infectious diseases. For ease of interpretation, we have the infectious disease equity market volatility index divided by 100.

	(1)	(2)
	OLS	Tobit
	Dividends/Total Assets	Dividends/Total Assets
		
**Infectious Disease Index**	-0.042***	-0.137***
	**(-8.055)**	**(-20.920)**
Ln (Total Assets)	0.000***	0.008***
	(3.079)	(71.551)
Leverage	0.002***	0.004***
	(2.610)	(9.069)
Profitability	0.001***	0.002***
	(2.600)	(3.396)
Capital Investments	-0.000	-0.025***
	(-1.583)	(-8.711)
Advertising Intensity	-0.006	-0.038***
	(-0.626)	(-4.425)
R&D Intensity	0.020***	0.044***
	(8.211)	(24.069)
Cash Holdings	0.000	-0.019***
	(0.039)	(-14.698)
Discretionary Spending	0.002***	0.006***
	(2.834)	(11.502)
Share Repurchases	0.060***	0.172***
	(8.088)	(23.873)
EPU Index	0.000***	0.000***
	(4.840)	(25.470)
Constant	0.011***	-0.087***
	(11.890)	(-17.513)
Industry Fixed Effects	Yes	Yes
Observations	287,150	287,151
Adjusted/Pseudo R-squared	0.028	0.121

Robust t-statistics in parentheses.

*** p<0.01

** p<0.05

* p<0.1.

### c. Oster’s (2019) [10] method for testing coefficient stability

To further strengthen our results, we used Oster’s [10] method which can be used to assess the robustness of our results from omitted variable bias [89]. Oster [10] proposed to calculate the size of the influence from omitted variables that could overturn the key conclusion of an analysis. For example, suppose one finds that a variable is statistically significant and positive in a regression. In this case, we would need to find how large the influence from omitted variables would be in order to overturn the key conclusion of the testing variable from positive and significant to statistically indistinguishable from zero. Using Oster’s [10] method on our regressions in Table 2, we found that the effect of the omitted variables would have to be more important than observed variables by 7.24 times in order to make the estimated negative effect of the infectious disease index be statistically indistinguishable from zero. In general, if the ratio is greater than one, the results are considered robust. Thus, this strengthens the robustness of our results. See Oster’s [10] for more detail about how to assess coefficient stability.

### d. Dynamic panel data analysis

Additionally, we executed a dynamic panel data analysis. In dynamic panel data estimation, variables are first-differenced to remove unobserved time-invariant confounding variables. However and by construction, the first-differenced specification cannot be estimated by OLS as the first-differenced lagged dependent variables are correlated with the error term. To overcome this, it is common to use the dynamic in the system and employ lag variables (both difference and level) of dependent and independent variables as instruments. It is also common to apply the Arellano-Bond test to check for the autocorrelation in the residuals and the Sargan-Hansen overidentifying restrictions test to check for instruments validity [90].

As commonly practiced in the literature, we started our analysis based on Arellano and Bond [91] difference GMM estimation and Arellano and Bover [92] system GMM estimation (based on “xtabond” and “xtabond2” commands in Stata). We found that the Arellano-Bond test for autocorrelation and Sargan-Hansen test for overidentifying restrictions are invalid. A unique but problematic feature of the difference GMM estimation of Arellano and Bond [91] and Arellano and Bover [92] system GMM is the use of a large number of lags as instruments for the difference equation and the level equation [93,94] when the number of variables and time periods in the model specification increases. This can lead to overfitting which often results in the rejection of the Arellano-Bond and the Sargan-Hansen test.

To overcome the issue of “too many instruments” problem, we adopted a recently developed procedure of Kripfganz [94] (based on “xtdpdgmm” command in Stata). Two major advantages of this approach is that it allows users to select which variables to be included as instruments rather than using all possible lags (i.e., “curtailing”) as well as the ability to “collapse” the set of moment conditions. See Kripfganz [94] for more detain about “curtailing” and “collapsing”. Table 7 reports of our estimation based on the approach of Kripfganz [94]. Instead of using more than 1,000 instruments in our context when using Arellano and Bond [91] difference GMM estimation and Arellano and Bover [92] system GMM estimation, the approach of Kripfganz [94] allows us to use only 16 instruments in the estimation. Both the Arellano-Bond test for autocorrelation and Sargan-Hansen test for overidentifying restrictions are valid in this case. The coefficient of the infectious disease index remains negative and significant.

**Table 7:** Dynamic panel and overidentification restriction analysis. The set of instruments used in the difference equation are as follows: 1) contemporaneous values of Ln (Total Assets), Leverage, Profitability, R&D Intensity, Discretionary Spending, EPU Index, and the infectious disease equity market volatility as well as 2) contemporaneous and the first lag values of Capital Investments, Share Repurchases, Advertising Intensity, and Cash Holdings. We also included the second lag of Dividends/Total Assets as instruments in the difference equation as well. In total, there are 16 instruments.

	(1)
	Dividends/Total Assets
**Infectious Disease Index**	-0.055***
	**(-0.009)**
Ln (Total Assets)	0.001*
	(0.001)
Leverage	0.000
	(0.000)
Profitability	-0.001**
	(-0.000)
Capital Investments	-0.000
	(-0.000)
Advertising Intensity	0.018*
	(0.011)
R&D Intensity	0.016***
	(0.003)
Cash Holdings	0.005***
	(0.001)
Discretionary Spending	0.002***
	(0.000)
Share Repurchases	0.038***
	(0.005)
EPU Index	0.000***
	(0.000)
Dividends/Total Assets (t-1)	0.408***
	(0.069)
Dividends/Total Assets (t-2)	0.180**
	(0.073)
Observations	228,435
Arellano—Bond Test (1)	0.000
Arellano—Bond Test (2)	0.115
Sargan—Hansen Test (1)	0.505
Sargan—Hansen Test (2)	0.540

Robust t-statistics in parentheses.

*** p<0.01

** p<0.05

* p<0.1.

### e. The role of independent directors

In our context and based on agency theory, self-interested managers are less motivated to pay dividend since this will reduce the free cash flow that they could otherwise use for their own benefits [27]. This scenario is more likely in the presence of crisis induced by infectious diseases since managers may use pandemic as an excuse to cut dividends. Thus, a more effective governance mechanism such as board independence should reduce agency problems and reduce the ability of self-interested managers to cut dividends.

To provide further insight on this matter, we investigated the effect of independent directors on dividend policy in the presence of uncertainty induced by infectious diseases. To allow for the possibility of non-linear effect, we included the percentage of independent directors on the board as well as the interreact term of this variable and the infectious disease index. The regression results are shown in Table 8. The coefficient of the interaction terms both in Model 1 and Model 2 are positive and significant. The coefficient of the infectious disease index remains negative and significant. The findings is consistent with the possibility that the dividend cut in the presence of uncertainty induced by infectious diseases is partly motivated by agency problems. Consistent with findings in the literature (e.g., [27]), more effective governance alleviates the agency problems and forces managers to release more cash to shareholders.

**Table 8:** The effect of independent directors on dividends in the presence of infectious diseases. Our measure of disease-related uncertainty is the infectious disease equity market volatility developed by Baker et al. (2020) [9]. Using sophisticated textual analysis, Baker et al. (2020) [9] search for news articles related to infectious diseases and equity market volatility. A higher fraction of these news articles to all articles in each time period signifies a higher level of market uncertainty that can be ascribed to infectious diseases. For ease of interpretation, we have the infectious disease equity market volatility index divided by 100.

	(1)	(2)
	OLS	Tobit
	Dividends/Total Assets	Dividends/Total Assets
		
**Infectious Disease Index × % Independent Directors**	0.011**	0.014**
	**(2.276)**	**(2.119)**
Infectious Disease Index	-1.107***	-1.559***
	(-3.223)	(-3.194)
% Independent Directors	0.000	0.000
	(0.025)	(0.838)
Ln (Board Size)	0.012***	0.029***
	(6.754)	(20.056)
Ln (Total Assets)	0.000	0.002***
	(1.101)	(9.778)
Leverage	0.006**	0.004***
	(2.480)	(2.932)
Profitability	0.042***	0.084***
	(6.758)	(27.570)
Capital Investments	-0.007	-0.034***
	(-0.911)	(-5.615)
Advertising Intensity	0.048**	0.041***
	(2.030)	(4.030)
R&D Intensity	-0.015	-0.099***
	(-1.613)	(-13.193)
Cash Holdings	0.010***	-0.004*
	(2.897)	(-1.957)
Discretionary Spending	0.015***	0.022***
	(5.462)	(11.437)
Share Repurchases	0.020**	0.021***
	(2.263)	(3.410)
EPU Index	0.000*	0.000
	(1.819)	(0.169)
Constant	-0.027***	-0.060***
	(-5.743)	(-8.370)
Industry Fixed Effects	Yes	Yes
Observations	27,726	27,726
Adjusted R-squared	0.138	-0.149

Robust t-statistics in parentheses.

*** p<0.01

** p<0.05

* p<0.1.

## V. Conclusions

We relax Modigliani and Miller’s [18] assumption of perfect capital markets by introducing and examining the effect of the uncertainty associated with infectious diseases on corporate dividend policy. We used a unique text-based measure of infectious diseases that includes not only the Covid-19, but also other important diseases, such as SARs, MERs, and Ebola. Based on a sample of 287,151 firm-year observations that covers more than 30 years of data, our results show that higher uncertainty associated with infectious diseases significantly reduce dividends. Our findings are consistent with the precautionary hypothesis where companies exercise more caution during difficult times and reduce dividends.

To demonstrate that our findings are not driven by economic uncertainty in general, we controlled for the degree of economic policy uncertainty (EPU) index proposed by Baker et al. [9]. Our results are qualitatively unaffected by this inclusion. We executed additional robustness checks which include propensity score matching, entropy balancing, dynamic panel data analysis with over-identifying restriction tests, and Oster’s [10] approach for testing coefficient stability. All these robustness checks validate our results.

Finally, we explored how dividend policy is influenced by board independence. We concentrated on board independence since it is one of the most important mechanism for corporate governance. Our results show that having more independent directors reduces the effect of uncertainty associated with infectious diseases on corporate dividends. This implies that the effect of uncertainty associated with infectious diseases on corporate dividends is partly driven by agency problems which can be mitigated by more effective governance.

## Supporting information

S1 Appendix (DOCX)
