# Supplementary material for: Infectious diseases, dividend policy, and independent directors: Evidence from textual analysis
Source: PLoS One. 2023 Feb 2;18(2):e0281109. doi: 10.1371/journal.pone.0281109 (PMC9894484; doi:10.1371/journal.pone.0281109)
Supplement: S1 Appendix — (DOCX) [file pone.0281109.s001.docx]

**Appendix**

**Table A1: Variable definitions**

| Variable | Definition |
| --- | --- |
|  |  |
| Infectious Disease Index |  |
| Infectious disease Index | Our measure of disease-related uncertainty is the infectious disease |
|  | equity market volatility developed by Baker et al. (2020). Using |
|  | sophisticated textual analysis, Baker et al. (2020) search for news |
|  | Articles related to infectious diseases and equity market volatility |
|  | A higher fraction of these articles to all articles in each time period |
|  | signifies a higher level of uncertainty that can be attributed to |
|  | infectious diseases. |
| Dividend Variables |  |
| Dividends/Total Assets | Total Dividends divided by Total Assets |
| Dividends/Sales | Total Dividends divided by Sales |
| Dividends/Net Income | Total Dividends divided by Net Income |
| Firm-specific Characteristics |  |
| Firm Size; Ln (Total Assets) | Total Assets |
| Leverage | Total Debt/Total Assets |
| Profitability | EBIT/Total Assets |
| Capital Investments | Capital Expenditures/Total Assets |
| Advertising Intensity | Advertising Expense/Total Assets |
| R&D Intensity | R&D Expense/Total Assets |
| Cash Holdings | Cash Holdings/Total Assets |
| Discretionary Spending | SG&A Expense/Total Assets |
| Share repurchases | Share Repurchases/Total Assets |
| EPU Index | Economic policy uncertainty (EPU) index  proposed by Baker et al. [9] |
| Board Attributes |  |
| % Independent Directors | Percentage of Independent Directors on the Board |
| Board Size | The Number of Directors on the Board |
